# Influence of vitamin B auxotrophy on nitrogen metabolism in eukaryotic phytoplankton

**DOI:** 10.3389/fmicb.2012.00375

**Published:** 2012-10-19

**Authors:** Erin M. Bertrand, Andrew E. Allen

**Affiliations:** Department of Microbial and Environmental Genomics, J. Craig Venter InstituteSan Diego, CA, USA

**Keywords:** cobalamin, thiamine, S-adenosylmethionine, nitrogen, sulfur, urea cycle, microbial interactions, harmful algal blooms

## Abstract

While nitrogen availability is known to limit primary production in large parts of the ocean, vitamin starvation amongst eukaryotic phytoplankton is becoming increasingly recognized as an oceanographically relevant phenomenon. Cobalamin (B_12_) and thiamine (B_1_) auxotrophy are widespread throughout eukaryotic phytoplankton, with over 50% of cultured isolates requiring B_12_ and 20% requiring B_1_. The frequency of vitamin auxotrophy in harmful algal bloom species is even higher. Instances of colimitation between nitrogen and B vitamins have been observed in marine environments, and interactions between these nutrients have been shown to impact phytoplankton species composition. This review surveys available data, including relevant gene expression patterns, to evaluate the potential for interactive effects of nitrogen and vitamin B_12_ and B_1_ starvation in eukaryotic phytoplankton. B_12_ plays essential roles in amino acid and one-carbon metabolism, while B_1_ is important for primary carbohydrate and amino acid metabolism and likely useful as an anti-oxidant. Here we will focus on three potential metabolic interconnections between vitamin, nitrogen, and sulfur metabolism that may have ramifications for the role of vitamin and nitrogen scarcities in driving ocean productivity and species composition. These include: (1) B_12_, B_1_, and N starvation impacts on osmolyte and antioxidant production, (2) B_12_ and B_1_ starvation impacts on polyamine biosynthesis, and (3) influence of B_12_ and B_1_ starvation on the diatom urea cycle and amino acid recycling through impacts on the citric acid cycle. We evaluate evidence for these interconnections and identify oceanographic contexts in which each may impact rates of primary production and phytoplankton community composition. Major implications include that B_12_ and B_1_ deprivation may impair the ability of phytoplankton to recover from nitrogen starvation and that changes in vitamin and nitrogen availability may synergistically impact harmful algal bloom formation.

## Introduction

The rate, magnitude, and species composition of marine primary production has a profound influence of global carbon cycling and therefore climate. As a result, factors controlling the growth of marine primary producers are of considerable interest. While nitrogen and iron availability are often considered the primary bottom-up controls on short-term marine primary productivity, the importance of organic growth factors received considerable early attention (Cowey, 1956; Droop, 1957, 1962; Menzel and Spaeth, 1962; Provasoli, 1963; Gold, 1968; Carlucci and Silbernagel, 1969; Carlucci and Bowes, 1970; Swift and Taylor, 1972; Swift, 1981) and is the subject of renewed interest (e.g., Sañudo-Wilhelmy et al., 2012).

Recent developments in analytical techniques (Okbamichael and Sañudo-Wilhelmy, 2004; Sañudo-Wilhelmy et al., 2012), application of trace metal clean bioassay experiments (Panzeca et al., 2006; Sañudo-Wilhelmy et al., 2006; Bertrand et al., 2007; Gobler et al., 2007; Koch et al., 2011), and culture-based surveys of vitamin requirements (Croft et al., 2005; Tang et al., 2010) have identified B_12_ (cobalamin) and B_1_ (thiamine) as highly important growth factors for eukaryotic phytoplankton and suggest that these micronutrients have the potential to broadly influence marine productivity and species composition. Due to the fact that B_12_ and B_1_ both play numerous essential roles in cellular biochemistry, starvation for these nutrients has the potential to impact phytoplankton cellular metabolism through a range of mechanisms. The increase in available genome and transcriptome data for relevant organisms has opened doors for new modes of inquiry into the role of these micronutrients in phytoplankton metabolism as well as their potential for interaction with additional states of nutrient deprivation (Croft et al., 2006; Helliwell et al., 2011; Bertrand et al., 2012). Here we review available data to examine potential interactions between B_12_, B_1_, and nitrogen deprivation and sulfur metabolism in eukaryotic phytoplankton communities and provide insight into the potential implications of these interactions for phytoplankton evolutionary trajectories and biogeochemical cycling.

## Cobalamin and thiamine

### Production, demand, and biochemical function

Cobalamin, B_12_, is a cobalt-containing organometallic micronutrient that conducts elegant chemistry facilitated by the controlled reactivity of the axial Co-C bond in methyl and adenosylcobalamin (Schrauzer and Deutsch, 1969; Lexa and Savant, 1983; Drennan et al., 1994). The resulting reactivity provides the biochemical capacity for methylation and rearrangement reactions, where a hydrogen atom on one carbon constituent is exchanged for another functional group, typically a methyl, amine, or alcohol group. Cobalamin is believed to be produced only by select bacteria and archaea (Roth et al., 1996; Martens et al., 2002) and is required by humans and other metazoans, by an estimated half of all eukaryotic phytoplankton (Tang et al., 2010) and by some bacteria that are not able to synthesize it (Rodionov et al., 2003; Zhang et al., 2009). Vitamin B_12_ biosynthesis requires over 30 enzymatic steps and significant consumption of cellular energy, carbon, nitrogen, cobalt, zinc, and in some cases iron (Roth et al., 1996; Raux et al., 2000).

Vitamin B_12_ demand by eukaryotic phytoplankton is thought to arise from its role as a cofactor in the enzyme methionine synthase, which catalyzes the conversion of homocysteine and methyl-tetrahydrofolate to tetrahydrofolate and methionine (Table 1). The active form of B_12_ in methionine synthase is methylcobalamin. Algae that require B_12_ absolutely posses only the B_12_-dependant version of this enzyme (MetH), while those that do not have an absolute requirement have the ability to use an alternative B_12_ independent version (MetE) (Croft et al., 2005). Phylogenetic analysis of metE and metH coding sequences support a complex evolutionary history of *metE* gene gain and loss within eukaryotic organisms. In contrast, the phylogeny of *metH* is well resolved and apparently monophyletic in eukaryotes (Helliwell et al., 2011). These analyses suggest that absolute B_12_ requirements in eukaryotic algae have likely arisen as a result of multiple independent loses and acquisitions of *metE* from eukaryotic genomes. Indeed, under high B_12_ concentrations, *metH* is continually expressed by algal strains, whereas *metE*, if present, is repressed until B_12_ is depleted (Croft et al., 2005; Helliwell et al., 2011; Bertrand et al., 2012). These results suggest that B_12_ auxotrophy in eukaryotic algae arose as a function of variable B_12_ availability in the environment. This is supported by observations that the distribution of *metE* in eukaryotic phytoplankton does not follow phylogenetic lines. Importantly, there is strain level variability in whether or not phytoplankton exhibit an absolute requirement for B_12_ (Tang et al., 2010). In addition, B_12_ is a cofactor in the enzyme methylmalonyl coA mutase (mmcM), which is encoded in some but not all B_12_-requiring phytoplankton genomes (Table 1). *mmcM*'s function in eukaryotic phytoplankton remains somewhat unclear, though it likely plays a role in the citric acid cycle as well as fatty acid and propionate metabolism. However, the presence of *mmcM* genes in phytoplankton genomes does not confer a B_12_ requirement under typical laboratory growth conditions (Croft et al., 2006).

**Table 1:** **Vitamin B_12_-related genes in sequenced marine eukaryotic phytoplankton genomes and select marine prokaryotic genomes**.

	**Auran**	**Phatr**	**Thaps**	**Psemu**	**Fracy**	**Emihu**	**Ostta**	**Ostlu**	**MicPu**	**Chlre**	**ChlNC**	**Syn 8102**	**Pro 9313**	**P. ubique**
MetH	34875	23399	693	213031	207237	423073	16287	45056	148156	76715	36916	SYNW 1238	PMT0729	x
MetE	x	28056	x	x	228154	x	x	x	x	154307	141995	x	x	x
MmcM	26280	51830	33685	261420	273786	120906 417351	x	x	x	x	18280	x	x	x
CBA1	63075	48322	11697	235642	241429, 246327, 273295, 269995	x	x	x	x	x	x	x	x	x
RNR Class 2 B_12_	x	x	x	x	x	x	x	x	x	x^#^	x	SYNW 1147	PMT 0793	x
RNR Class 1 Fe; small	65685 59025	39306 17523	32555 8522 3367	67342 252139	268008 206256	470988 469622 200748	22908 8886	32923 39468	155636 174818	188785 144621	34102 10712 57791	x	x	PB7211_302
RNR Class 1 Fe; Large	30730, 37557, 24558	42726, 45529	370, 268807	223844 319245	260490, 262570, 205957	449248, 212824	22667	48569	167892	185583	32953	x	x	PU1002_00625
B_12_ biosynthesis	No	No	No	No	No	No	No	No	No	No	No	Yes	Yes	No
*B_12_ Aux. by genome*	Yes	No	Yes	Yes	No	Yes	Yes	Yes	Yes	No	No	No	No	No
*B_12_ Aux. by culture*	Yes (Tang et al., 2010)	No (Droop, 1958)	Yes (Guillard and Ryther, 1962)	Yes (Tang et al., 2010)^*^	No (Helliwell et al., 2011)	See (Helliwell et al., 2011)	Yes (Helliwell et al., 2011)		Yes (Helliwell et al., 2011)	No (Provasoli and Carlucci, 1974)	No (Shihira and Krauss, 1965)			

The hypothesized vitamin requirements of each strain are also given, along with whether culture-based confirmation of auxotophic status is available. This table represents an expansion of information given in Croft et al., 2006 and Helliwell et al., 2011. No eukaryote is known to make vitamin B_12_; B_12_ auxotrophy in eukaryotic algae appears to depend on the presence or absence of B_12_-independent methionine synthase (Croft et al., 2005; Helliwell et al., 2011). Auran, Aureococcus anophagefferans; Phatr, Phaeodactylum tricornutum; Thaps, Thalassiosira pseudonanna; Psemu, Pseudo-nitzschia multiseries CLN-47; Fracyl, Fragilariopsis cylindrus; Chlre, Chlamydomonas reinhardtii (v4; filtered or best proteins); Emihu, Emiliania huxleyi; Ostta, Ostreococcus taurii; Ostlu, Ostreococcus lucimarinus V2 filtered model proteins; MicPu, Micromonas pusilla CCMP1545 c3.0, filtered model proteins; ChlNC, Chlorella sp. NC64A filtered proteins; Syn8102, Synechococcus sp. WH8102; Pro9313, Prochlorococcus marinus MIT 9313; P. ubique, Candidatus Pelagibacter ubique SAR11 HTCC1002.

* Auxotrophy tested in culture of a different strain.

# Has a protein with substantial sequence similarity but missing active site: (154521).

Thiamine, B_1_, is a cofactor required by all organisms and produced by many prokaryotes as well as by fungi, plants, and some eukaryotic phytoplankton (Webb et al., 2007). It is a sulfur-containing compound, produced though joining of a pyrimidine and a thiazole moiety, and is phosphorylated in its coenzyme form (thiamine diphosphate). In bacterial biosynthetic pathways, thiazole biosynthesis requires six distinct enzymatic steps and pyrimidine synthesis requires two (Rodionov et al., 2002; Jurgenson et al., 2009). While the bacterial thiamine biosynthesis pathway is well characterized, eukaryotic biosynthesis pathways remain poorly understood and appear to be distinct in plants and fungi (Jurgenson et al., 2009). Algal thiamine biosynthesis is even less well-characterized but likely conducted by some enzymes similar to bacterial thiamine biosynthesis genes and some enzymes similar to the yeast and plant pathways (Croft et al., 2006), though this remains to be conclusively demonstrated.

While thiamine was one of the first organic cofactors identified as important for algal growth, early work showed that there are some phytoplankton strains that produce thiamine *de novo*, and some that can scavenge and salvage either the thiazole or pyrimidine moieties from the environment in order to construct a functional cofactor (Droop, 1958; Provasoli and Carlucci, 1974). Preliminary inquiry into eukaryotic phytoplankton genomes conducted via identification of coding sequences similar to those encoding known bacterial, fungi, and plant thiamine biosynthesis enzymes suggests that there are potentially different pathways for thiamine production in stramenopiles versus the green algal lineage (Table 2, McRose et al., 2012). The absence of a gene encoding ThiC, a protein involved in pyrimidine biosynthesis, appears to correlate with B_1_ auxotrophy in algae with sequenced genomes, regardless of lineage (Table 2). This intriguing observation warrants further exploration. Since ThiC is involved in pyrimidine biosynthesis, the relationship between ThiC gene presence and thiamine auxotrophy is likely to hold only for auxotrophs with the ability to synthesize thiamine diphosphate when provided the pyrimidine moiety, not those that can synthesize the vitamin when provided with the thiazole moiety, such as some dinoflagellates and cryptophytes (Droop, 1958). ThiC is an interesting protein; it requires S-adenosyl methionine (SAM) for activity (Chatterjee et al., 2008), is an iron-sulfur cluster protein, and is present in both the plant and bacterial thiamine biosynthesis pathways (Goyer, 2010).

**Table 2:** **Vitamin B_1_ (Thiamine)-related genes in sequenced eukaryotic phytoplankton genomes**.

	**Auran**	**Phatr**	**Thaps**	**Psemu**	**Fracy**	**Emihu**	**Ostta**	**Ostlu**	**MicPu 1545**	**MicPu 299**	**Chlre**	**ChlNC**
ThiC	x	38085	41733	255053	225659	x	x	x	x	x	192720	136333
thiD+thiE/Thi6/TenI	x	47293	262964-3	320126	153126, 161112	102278	20618, 6224	17535	52893	x	390684	58425
ThiF^*^	31873, 32858	34373, 20318	261602, 35049	207357, 293997	194811, 275015	68584	19906	38170	51160	113992	138485	22673
dsx	59650	bd1689	574	65889	206898	440786	15650	48774	121145	107366	196568	59788
ThiG^%^	x	PhtrCp129	ThpsCp126, bd1620	–	Scaffold 95, 27066–27869	Emhu Cp072	x	–	x	–	x	x
ThiS^%^	AuanCp078	PhtrCp091	ThpsCp091	–	Scaffold 95, 5640–5849	x	x	–	x	–	x	x
ThiO/H^**^	72208	31544	263655	230060	241529	53832	x	x	x	x	196226	30311
Thi4	x	x	x	x	x	x	20276^∧^	x	52894^∧^	x	185190	22703
TPK	20636	5423	262503^@^	264355	86232	56054	10431	12109	163134	109022	72868	11702
ThiM/10	x	x	x	x	x	x	x	x	x	x	126905^$^	53510
*B1 Aux. by culture*	Yes (Tang et al., 2010)	No (Droop, 1958)	No (Guillard and Ryther, 1962)	No (Tang et al., 2010)^***^	No (Bertrand, unpublished)	Yes (Carlucci and Bowes, 1970)^***^			Yes (McRose et al., 2012)	Yes (McRose et al., 2012)	No (Provasoli and Carlucci, 1974)	No (Shihira and Krauss, 1965)

The hypothesized vitamin requirements of each strain are also given, along with whether culture-based confirmation of auxotophic status is available. This table expands information given in Croft et al., (2006). Auran = Aureococcus anophagefferans; Phatr = Phaeodactylum tricornutum; Thaps = Thalassiosira pseudonanna; Psemu = Pseudo-nitzschia multiseries CLN-47; Fracyl = Fragilariopsis cylindrus; Chlre = Chlamydomonas reinhardtii (v14; filtered or best proteins); Emihu = Emiliania huxleyi; Ostta = Ostreococcus taurii; Ostlu = Ostreococcus lucimarinus V2 filtered model proteins; MicPu = Micromonas pusilla CCMP1545 c3.0, filtered model proteins; ChlNC = Chlorella sp. NC64A filtered proteins.

X = not found, “–” = search not possible (chloroplast genome not available)

* ThiF is not easily assigned because of similarities with MoeB/Z

% ThiG and ThiS are often chloroplast encoded

∧ unclear, potential Thi4 (similarity to tenA proteins too)

@ uncertain assignment

$ mutants of this are thiamine auxotrophs

** The diatoms appear to have ThiO, Chlre, and ChlNC have thiH

*** Auxotrophy tested in culture of a different strain.

Thiamine catalyzes a number of transformations that are important in carbohydrate and branched amino acid metabolism including those involved in glycolysis, the pentose phosphate pathway, and the tricarboxylic acid pathway. These notably include 2-oxoglutarate dehydrogenase (ODG), pyruvate dehydrogenase/decarboxylase, branched-chain α-ketoacid dehydrogenase, as well as transketolases, acetolactate synthase, and alpha-keto acid dehydrogenase. The chemistry involved in these reactions often includes two carbon group transfers or dehydrogenation reactions (Frank et al., 2007). There is mounting evidence that thiamine may play additional, non-cofactor roles as well. In plants, thiamine has been tied to cellular responses to oxidative stress and disease. Plants subjected to hydrogen peroxide, salt stress, and high light stress, for example, all showed enhanced thiamine production and increased thiamine biosynthesis protein transcripts, such as ThiC (reviewed in Goyer, 2010; Rapala-Kozik et al., 2012). It is possible that this increase in thiamine under stress results from demand for transketolase activity in the pentose phosphate pathway which regenerates NADPH required for activity of some antioxidants (Goyer, 2010). However it is also possible that thiamine itself functions as an antioxidant in these cells, as thiamine compounds have antioxidant capacities, likely through the transfer of H+ from amino groups on the thiazole and pyrimidine rings to reactive species (Hu et al., 1995; Lukienko et al., 2000; Bettendorff and Wins, 2009). While there has been comparatively little study of these potential roles of thiamine in algae, available evidence suggests that thiamine biosynthesis per cell in diatoms increases as a function of increasing cell density and nutrient depletion, which may be caused by the increase in oxidative stress (Pinto et al., 2003).

Thiamine auxotrophy is strikingly different from B_12_ auxotrophy in algae; while B_12_ requirements are determined by the ability of a phytoplankton strain to replace B_12_-requiring metabolisms (Table 1), B_1_ auxotrophy is defined by whether or not an algal strain is able to synthesize the vitamin *de novo* (Table 2). Considering that the enzymes for B_1_ biosynthesis are not yet completely elucidated in algae, it is difficult to discern, through analysis of protein coding sequences, the evolutionary origin of B_1_ auxotrophy. However, observations concerning the phylogenetic distribution of thiamine auxotrophy support the notion that biosynthesis potential may have also been lost and acquired multiple times. For instance, in the case of two strains of the same species of dinoflagellate, isolated from the same site, one is a B_1_ auxotroph and one is not (Tang et al., 2010). Among *Micromonas* spp. strains with thiamine requirements, one is missing more of the biosynthetic pathway than the other (McRose et al., 2012; Table 2). These data suggests that like B_12_, B_1_ auxotrophy in algae has likely arisen numerous times though gene loss events. Such loss events could be driven by chronically high thiamine availability coupled to transcriptional repression and associated loss of purifying selection and gene erosion. While this repression is yet to be documented, eukaryotic phytoplankton genomes encode thiamine riboswitches (Croft et al., 2007; Worden et al., 2009); which offer a mechanism by which high thiamine bioavailability can regulate gene transcription.

### Oceanographic distributions and cycling

In the ocean, dissolved (0.2 μm) vitamin B_12_ and B_1_ show variable but often nutrient-like depth profiles and are thought to be present in sub-picomolar quantities to up to 30 pM for B_12_ and 500 pM for B_1_ (Sañudo-Wilhelmy et al., 2012). Concentrations of these vitamins in coastal waters are generally higher than in open ocean regions (Panzeca et al., 2009). Measurement techniques for B vitamins in seawater remained restricted to bioassays (Menzel and Spaeth, 1962; Carlucci, 1966) until solid phase extraction, high pressure liquid chromatography methods were developed (Okbamichael and Sañudo-Wilhelmy, 2004; Okbamichael and Sanudo-Wilhelmy, 2005). Development of these techniques, coupled with mass spectrometry, has fostered more efficient and accurate methods for vitamin detection and quantitation in seawater. Such methods however still require inconveniently large volumes and are not currently optimized to detect B_12_ with different α or β axial groups or differentially phosphorylated forms of thiamine, which may be present in seawater and could be important for bioactivity as well as biogeochemical cycling. In addition, concentration measurements alone may not be an informative measure of the impact of vitamins on marine biogeochemical processes since their concentrations are low and they may be cycled and regenerated rapidly in the euphotic zone as a function of biological production and consumption as well as abiotic processing. The halflife of B_12_ in the surface ocean with respect to photodegradation alone is approximately 4 days, while B_1_ is more resistant to abiotic transformations in seawater (Gold et al., 1966; Carlucci et al., 1969). This, along with differences in production and consumption of vitamins by different components of marine microbial communities, may explain the observation that B_12_ and B_1_ concentrations and cycling may be decoupled in the water column (Panzeca et al., 2008; Sañudo-Wilhelmy et al., 2012). It remains a challenge to reconcile the interesting observation that B vitamin abundance patterns are associated with basin-scale water mass origin (Sañudo-Wilhelmy et al., 2012) with the likely rapid changes in production and consumption of these vitamins. To address this question, continued efforts to measure these vitamins, along with assessments of microbial community composition and vitamin acquisition rates, should include assessments of variability on short (hours to days) as well as seasonal timescales.

Either through dissolved organic matter exudation and cell lysis via the cycling of the microbial loop, (Azam, 1998; Karl, 2002; Droop, 2007) or through direct symbiotic interaction (Croft et al., 2005), some portion of the bacterial and archaeal community must be the ultimate source of vitamin B_12_ to eukaryotic phytoplankton. The genetic potential for vitamin B_12_ production remains largely uncharacterized in any marine environment (Bertrand et al., 2011a). This is in part because the occurrence of the biosynthesis pathway among bacterial and archaeal lineages is extremely variable and is not easily queried using typical phylogenetic profiling techniques. An exception to this is the marine cyanobacteria, where all sequenced genomes appear to contain the B_12_ biosynthetic pathway (Rodionov et al., 2003), and numerous strains have been shown to produce significant amounts of B_12_ (Bonnet et al., 2010). The identity of other groups that contribute significantly to oceanic B_12_ production remains unclear, however, and is of particular importance in regions with scarce cyanobacterial populations such as the polar oceans (Caron et al., 2000; Marchant, 2005). The extremely abundant SAR11 group appears to neither synthesize nor require the vitamin (Table 1). In addition, there are examples from many sequenced marine bacterioplankton genomes of strains that either cannot produce the vitamin themselves but require it for various metabolisms, or those that can salvage degraded B_12_ for repair and reuse (Bertrand et al., 2011a). In sum, B_12_ uptake by marine bacteria and archaea can be as significant as uptake by eukaryotic phytoplankton (Bertrand et al., 2007; Koch et al., 2011). This results in a scenario in which eukaryotic phytoplankton likely compete for B_12_ resources with some components of the prokaryotic community (Bertrand et al., 2011b; Sañudo-Wilhelmy et al., 2012).

Thiamine sources to eukaryotic phytoplankton include *de novo* production, uptake or salvage from bacterial production, or uptake and salvage of thiamine produced by other algae (Carlucci and Bowes, 1970; Provasoli and Carlucci, 1974). Similar to B_12_, competition likely occurs for B_1_ amongst microalgae as well as between algal and bacterial groups since not all prokaryotes have the ability to produce thiamine (Rodionov et al., 2002). It remains unclear, however, what the relative importance of these uptake vectors are and how this varies across oceanic regions. A striking difference between B_12_ and B_1_ auxotropy is that B_1_ requirements in algae could potentially be supplied by growth with B_1_ producing algal strains as well as with some bacteria (Table 2). This is in contrast to B_12_ where the only potential source of B_12_ to auxotrophic algae is bacterial and archaeal production. This opens interesting avenues for exploration of species succession and potential commensalism between not only algae and bacteria but also between different algal strains.

**Figure 1:**
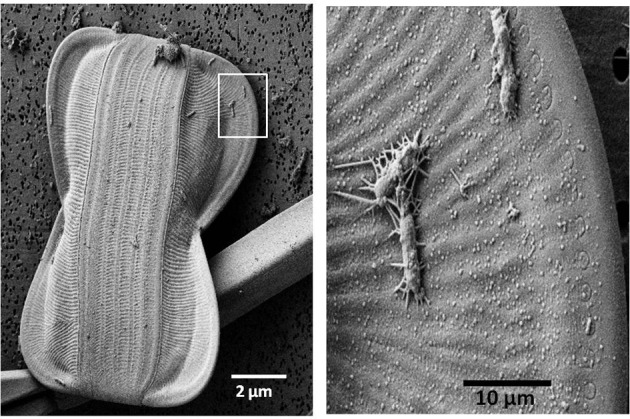
**Bacteria can be intimately associated with diatoms.** This sea ice *Amphiprora* diatom cell has bacterial cells attached through an apparently tight association likely via the use extracellular polymeric substances (EPS). SEM micrographs were collected at the UC Riverside Center for Nanoscale Science and Engineering. Samples were filtered, critical point dried to preserve cellular structures, coated with Pt:Pd to prevent charging, and imaged at 2 kv on a Zeiss 1540 FE-SEM.

Bottle incubation bioassay experiments have suggested that availability of B_12_ and to some degree B_1_ influence overall rates of primary production as well as phytoplankton community composition in regions ranging from the Southern Ocean to temperate coastal environments (Panzeca et al., 2006; Sañudo-Wilhelmy et al., 2006; Bertrand et al., 2007; Gobler et al., 2007; Koch et al., 2011). In many cases, addition of B vitamins to communities resulted in the proliferation of diatoms or dinoflagellates and larger groups of eukaryotic phytoplankton (Table 3). This may have important implications for carbon and nitrogen export as well as silica cycling, since larger phytoplankton tend to support a higher percentage of organic matter export. In addition, coastal and open ocean North Atlantic studies revealed that regions with higher B_12_ concentrations correlated with regions with high bacterioplankton productivity or density (Gobler et al., 2007; Panzeca et al., 2008), though it remains unclear whether these correlations are due to bacterial production of the vitamin or enhanced bacterial abundance as a result of higher B_12_ availability. In the Ross Sea of the Southern Ocean, bacterial abundance was shown to be low where primary production was stimulated by B_12_, meaning that where bacterioplankton communities were more numerous, B_12_ was less likely to limit primary production (Bertrand et al., 2011b). This suggests that bacterioplankton have an important impact on B_12_ supply to eukaryotic phytoplankton, at least in polar regions. However, intimate associations between bacteria and eukaryotic phytoplankton are known to occur (Figure 1; Cole, 1982; Grossart et al., 2005); the importance of these associations to B vitamin cycling and availability to phytoplankton in the marine environment are just beginning to be explored and offer numerous exciting avenues for continued research.

**Table 3:** **Results of B-vitamin supplementation in published marine bottle incubation bioassays**.

**Location**	**Experiments with stimulation of Chl a production by a B vitamin**	**B vitamin changed community composition?**	**Size class or functional group with biggest response**	**Notes**	**Interactions with N**	**References**
Long Island embayments	1/1	1/1	>5 μm	Observed correlation between dissolved B_12_, B_12_ drawdown and growth of large phytoplankton	Yes	Sañudo-Wilhelmy et al., 2006
Antarctic Peninsula	1/1	1/1	nd	Primary and secondary limitation by B_1_ + B_12_	nd	Panzeca et al., 2006
Ross Sea	2/3	3/3	Diatoms	–	nd	Bertrand et al., 2007
Long Island embayments	4/14	–	>5 μm	Fall experiments: large size fraction B vitamin limited	Yes	Gobler et al., 2007
Ross Sea	2/5	5/5	Diatoms	B_12_ uptake rates Fe limited	nd	Bertrand et al., 2011a,b
Gulf of Alaska	1/2	2/2	Dinoglagellates in coastal, diatoms in upwelling	N and Fe co-limitation with B_12_	Yes	Koch et al., 2011

nd = no data.

## The importance of nitrogen to eukaryotic phytoplankton

Nitrogen is an essential component of all life. The availability of nitrogen is thought to limit the productivity of marine microbial communities in large portions of the ocean (McCarthy and Carpenter, 1983; Hecky and Killam, 1988; Moore et al., 2004). Oceanic dissolved nitrogen distributions are driven in large part by coupled biological processing and large scale patterns in ocean circulation. Dissolved inorganic nitrogen (DIN) is generally considered to be the major source of nitrogen to marine microbial communities; the availability of these compounds (nitrate, nitrite, and ammonia), particularly in the oligotrophic ocean, can be depleted below 0.03 μM (Capone, 2000). Phytoplankton growth limitation by inorganic nitrogen availability has also been observed in coastal and upwelling environments (Kudela and Dugdale, 2000). The availability of this inorganic nitrogen has long been used, via nitrogen balancing calculations, to estimate organic matter export from the surface ocean (Eppley and Peterson, 1979), a concept which has profoundly influenced the field of biogeochemical oceanography. Models of the role of different nitrogen sources to phytoplankton and their microbial transformations have evolved to include additional processes, yet this conceptualization of balance between dissolve nitrate upwelled into the euphotic zone and export of biogenic and dissolved organic nitrogen (Bronk et al., 1994) continues to shape our understanding of controls on marine primary production and carbon cycling.

The relative availability of different N sources is now known to play a role in structuring phytoplankton species composition. Though reduced N compounds require less energy to assimilate, there are differences between taxonomic groups in terms of the impact of these differences on growth rate and the impact of ammonia availability on oxidized N acquisition (Dortch, 1990). In addition, differences in the ability of varying phytoplankton functional groups to respond to variable nitrogen concentrations and sources can create important niche dimensions. For instance, diatoms are a particularly successful group of eukaryotic phytoplankton that tend to dominate in coastal and upwelling regions. These locations are often characterized by highly variable nitrogen sources and concentrations. The ability of diatoms to respond quickly to pulsed nitrogen additions can, in part, explain a portion of their success in such environments. Their successful responses to these pulsed additions are partially explained by their ability to tightly couple anabolic and catabolic nitrogen transformations through incorporation of a complete urea cycle into central metabolism (Allen et al., 2011). Diatoms also tend to exhibit their maximal growth rates when grown on reduced nitrogen sources such as ammonia and urea (Dortch, 1990; Bender et al., 2012), but also in some cases dominate environments when nitrate is the dominant source of DIN. Their ability to take up and flexibly utilize a range of nitrogen sources also likely contributes to their role as a dominant phytoplankton group. We suggest that B vitamin deprivation may impair the ability of diatoms to effectively respond to and recover from nitrogen deprivation and that this may have important implications for interactions between marine microbial groups. This results from the fact that metabolisms impacted by B_1_ and B_12_ have important roles in pathways and mechanisms for allocation of cellular N recovery from N starvation.

## B vitamin and N interactions in oceanic environments

It is clear that marine bacterial communities, in some cases, compete with eukaryotic phytoplankton for inorganic nitrogen sources, including nitrate (Kirchman and Wheeler, 1998; Kirchman, 2000; Allen et al., 2001, 2005). These heterotrophic bacterial communities also conduct the canonical transformation of organic N sources to ammonia and dissolved organic nitrogen via cycling within the microbial loop. As a result, bacterial communities can be either net sources or net sinks of available N to phytoplankton communities (Kirchman, 2000; Zehr and Ward, 2002). This may vary as a function of the C:N ratio of available organic matter as well as the community composition of microbial assemblages (Kirchman, 2000). There are clear parallels between N and B vitamin availability in the ocean; the interaction between marine microbial groups plays a key role in shaping the influence these chemicals have on productivity. As a result, the implications of combined nitrogen starvation and B vitamin deprivation for eukaryotic phytoplankton will clearly be interactively impacted by bacterial communities. Intimately associated bacterial communities, such as those shown in Figure 1, have the potential to impact vitamin availability as well as nitrogen resources to phytoplankton; interactions between B vitamin and N dynamics in algal bacterial associations have yet to be explored, but are intriguing areas for research.

There have been two studies examining interactive impact of DIN and B vitamin addition on phytoplankton communities. In Long Island embayments, shifts from dinoflagellate dominated, primarily N limited communities in summer to diatom dominated blooms in fall coincided with decreases in B_12_ and B_1_ availability and increases in chlorophyll production upon B vitamin additions, suggesting that N and B vitamin availability both influence coastal phytoplankton species succession and biomass. Interestingly, in several instances, B_12_ or B_1_ and nitrate, when added together, stimulated chlorophyll production to a greater degree than adding either nutrient alone (Gobler et al., 2007). This interactive effect has yet to be mechanistically explored, but could be a function of vitamins being independently secondarily limiting, or could be explained by biochemical interactions between nitrogen and B vitamin production or demand (Saito et al., 2008). In a series of bottle incubation studies in the coastal, nitrogen limited region of the Gulf of Alaska, the addition of nitrate alone yielded enhanced productivity, and a shift from a dinoflagellate to diatom dominated community. In contrast, the addition of B_12_ and nitrate together yielded a community dominated by dinoflagellates (Koch et al., 2011). This striking result suggests that B vitamin availability severely impacted the response of the coastal phytoplankton community to nitrogen availability. This response suggests that the dinoflagellate community could not respond to nitrogen addition under B_12_ starvation conditions, either due to secondary, independent limitation of dinoflagellate growth by B_12_ availability or due to biochemical interactions between nitrogen and B_12_ metabolism leading to colimitation. Since a higher proportion of dinoflagellates are B_12_ auxtrophs (90%) than are diatoms (60%) (Tang et al., 2010), this response may be expected. However, diatom ability to respond to nitrogen additions over dinoflagellates under low B_12_ availability may not be entirely explained by differences in auxotrophy and warrants further exploration. Here we examine potential biochemical mechanisms for interaction between B vitamin and nitrogen metabolism.

## Molecular responses of eukaryotic phytoplankton to B vitamin starvation

**Figure 2:**
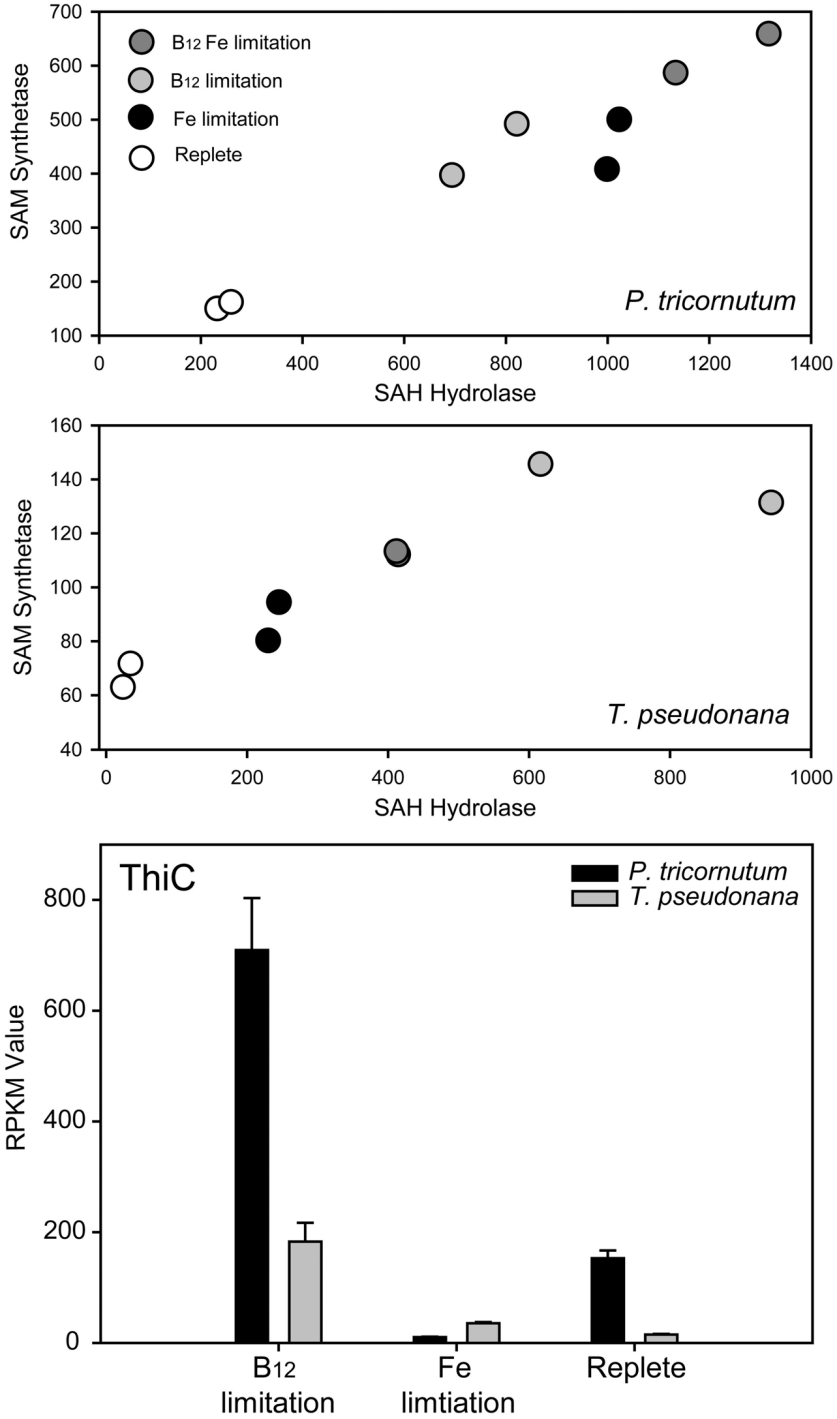
**Evidence from Bertrand et al., 2012 that AdoMet SAM starvation is an important consequence of B_12_ deprivation, with implications for thiamine biosynthesis.** SAM synthetase (Tp 39946, Pt 18319) converts methionine and ATP to SAM. SAM, after use for methylation reactions, is converted to S-adenosylhomocysteine (SAH). SAH can act as an inhibitor to methylation reactions because of its high affinity for most methyltranserfases. SAH hydrolase (Tp 28496; Pt bd 913) catalyzes the reversible interconversion of SAH to homocysteine and adenosine. The expression of the genes encoding these proteins in two diatoms appears to correlate. RPKM (Reads Per Kilobase of exon model per Million mapped reads) gene expression values are plotted against each other for each of eight samples in two diatoms, duplicates of replete, low B_12_, low B_12_ with low iron, and low iron alone. Expression under iron limited conditions was examined along with B_12_ to verify whether changes induced were likely a general stress response or more specific to the vitamin. In both diatoms, cells grown under nutrient replete conditions express these genes at the lowest level. Iron and B_12_ availability both influence the expression of these genes, with B_12_ having a greater impact of gene expression the B_12_ requiring diatom *T. pseudonana*. ThiC is a SAM-dependent protein required for pyrimidine moiety synthesis in thiamine biosynthesis. The expression of genes encoding ThiC in both these diatoms is elevated under low B_12_ availability and not under low iron availability, suggesting that thiamine biosynthesis, and B_12_ availability may be linked in these diatoms, potentially through B_12_ impacts on SAM availability.

There is some information available concerning the molecular responses of eukaryotic phytoplankton to B-vitamin starvation. While studies that examine the response of phytoplankton to B_1_ deprivation have not been described in detail (McRose et al., 2012), there has been comparatively extensive inquiry into the molecular response of phytoplankton to B_12_ deprivation. An important consequence of B_12_ deprivation in eukaryotic phytoplankton appears to be impaired methionine synthase activity and the use of B_12_-independant MetE over dependent MetH (Croft et al., 2005; Helliwell et al., 2011; Bertrand et al., 2012). Methionine serves not only as a protein-building amino acid but as the precursor to S-adenosylmethionine (AdoMet or SAM), an important methylating agent, propylamine donor, and radical source. Indeed, there is evidence that SAM deprivation is an important consequence of low B_12_ availability in diatoms (Bertrand et al., 2012; Figure 2). Notably, ThiC, an important algal thiamine biosynthesis protein, is SAM-dependant and responds to B_12_ deprivation in diatom cultures, suggesting that there may be consequences of B_12_ deprivation for thiamine production. In addition, dinoflagellate transcriptome and metatranscriptome sequencing studies reveal that SAM cycling genes are among the most highly expressed transcripts in multiple dinoflagellate species (Lidie et al., 2005; Moustafa et al., 2010; Toulza et al., 2010). These data, along with the high percentage of surveyed dinoflagellates that exhibit an obligate B_12_ requirement (>90%; Tang et al., 2010) suggest that B_12_ availability may have important implications for dinoflagellate SAM metabolism; perhaps a disproportionately important process relative to diatoms. This could potentially result from extensive dinoflagellate DNA methylation or increased demand due to toxin production, which has a high SAM requirement (Lin, 2011). In addition, impaired methionine synthase activity prevents efficient folate recycling, which has important implications for nucleic acid biosynthesis (Scott and Weir, 1981; Croft et al., 2005). Molecular evidence for altered folate metabolism has also been documented as a significant component of the diatom response to B_12_ deprivation (Bertrand et al., 2012). This likely holds true for other algal groups as well since the diatom response is similar to distantly related organisms such as humans and other metazoans (e.g., Scott and Weir, 1981).

Molecular aspects of acquisition of these vitamins in eukaryotic algae remains poorly understood. An important result of these inquiries into the molecular response of algae to vitamin deprivation has been the identification of proteins that are potentially involved in B_12_ or B_1_ acquisition. Bertrand et al. (2012) identified a previously uncharacterized protein, deemed CBA1, that is directly involved in B_12_ acquisition by diatoms and that is much more abundant in diatoms when they are experiencing B_12_ deprivation. This protein, however, appears to be restricted to the stramenopile lineage, suggesting that other eukaryotic algal groups utilize different, as of yet unidentified, pathways for B_12_ uptake (Bertrand et al., 2012). Several candidate proteins involved in thiamine trafficking have been identified in whole genome sequencing projects (Worden et al., 2009) and transcriptomic analyses of *Micromonas* cultures under thiamine deprivation have also resulted in identification of additional putative thiamine transporters in this B_1_ auxotrophic group (McRose et al., 2012).

**Figure 3:**
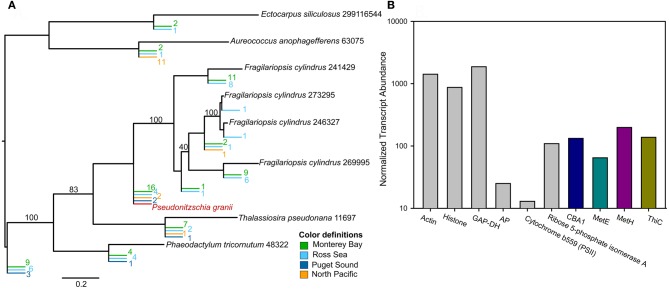
**The abundance and diversity of CBA1, B_12_ starvation indicator, and the abundance of ThiC, MetH, and MetE transcripts attributable to diatoms in Antarctic transcriptomic datasets, compared to the expression of transcripts encoding other well-characterized proteins (modified from Bertrand et al., 2012). (A)** Phylogenetic tree containing CBA1 sequences from 454 metatranscriptomic (cDNA) libraries from the Ross Sea of the Southern Ocean, Monterey Bay, Puget Sound, and the North Pacific. Reference sequences from *Phaeodactylum tricornutum*, *Fragilariopsis cylindrus*, *Thalassiosira pseudonana, Aureococcus anophagefferenas*, and *Ectocarpus siliculosus* genomes were used to construct these trees and are shown in black. CBA1-like sequences from environmental samples are shown in color, as described in the key. CBA1 transcripts were detectable in diverse marine environments, suggesting that cobalamin acquisition is an important component of diatom molecular physiology. **(B)** The normalized abundance of Open Reading Frames (ORFs) assigned to CBA1 from within the Ross Sea is shown in blue, MetE: PF01717 is shown in green, MetH:PF02965 is shown in purple, ThiC:PF01964 in yellow, while the abundance of read counts assigned to diatom ORFs containing well-characterized pfam domains for comparison [Actin: PF00022, Histone:PF00125, GAP-DH: PF02800, Alkaline Phosphatase: PF00245, Flavodoxin: PF00258, Cytochrome b559 (PSII): PF00283, Ribose 5-phosphate isomerase A: PF06026] are shown in gray. Read counts for each ORF where summed across six libraries from Ross Sea samples and RPKM values were calculated. RPKMs were then summed across all diatom ORFs that contained that a domain of interest. CBA1, MetE, MetH, and ThiC are not among the extremely abundant transcripts (e.g., those encoding Actin, GAP-DH, Histone) but are comparable to those encoding Calvin Cycle protein Ribose 5-phosphate isomerase A, and are more abundant than the transcripts encoding a cytochrome required for photosystem II activity (b559) as well as alkaline phosphatase (AP), suggesting that they are of importance to the molecular physiology of natural diatom communities.

Examination of transcripts encoding CBA1, ThiC, MetE, and MetH in natural Antarctic diatom communities revealed that all these transcripts are relatively abundant and therefore that B vitamin metabolism is likely an important component of the molecular physiology of field communities (Figure 3). These expression patterns suggest that Antarctic diatom communities are experiencing B_12_ deprivation (expressing MetE and CBA1) and that some subset of the community is able to potentially utilize B_12_ for methionine regeneration (MetH expression). This suggests that there are potentially different B_12_ quotas for different diatom species, with species experiencing starvation at varying intersections of cellular demand relative to ambient B_12_ availability. Additionally, there may be a subset of diatoms with localized B_12_ sources, such as intimately attached bacteria (Figure 1). These results suggest, however, that examining the distribution of B_12_—responsive transcripts in field populations will yield important insights into the impact of vitamin availability on community structure and primary productivity. For these analyses, it would be useful to know what percentage of the eukaryotic phytoplankton community possesses the ability to produce MetE; this would allow for more extensive interpretation of MetE and MetH transcript expression patterns. Similar analyses may be possible with thiamine—responsive genes in the future. Potential candidates for this include the recently identified putative transporters as well as ThiC, which appears to be present in genomes that are not auxotrophs and absent from genomes of organisms that require exogenous thiamine (Table 2).

## Potential B_12_ and B_1_ metabolic interactions with nitrogen in eukaryotic phytoplankton

### B vitamin and N starvation impacts on osmolyte production and utilization

Osmolytes are molecules that serve roles in osmoregulation. In eukaryotic phytoplankton, these include proline, glycine betaine (GBT), dimethylsulfonium propionate (DMSP), homarine, and isethionic acid (Boroujerdi et al., 2012). There are potentially important roles for B_12_, B_1_, methionine, SAM, and nitrogen metabolism in osmolyte production that likely result in interactive biochemical effects. One example is that methionine and SAM are both required for DMSP production, which is used by a subset of diatoms possibly as a cryoprotectant, osmolyte (Stefels, 2000), or antioxidant (Sunda et al., 2002), and is the precursor to the climatically important gas dimethylsulfide (Charlson et al., 1987). SAM recycling genes appear to play a role in the response of diatoms to low nitrogen, suggesting that there may be synergistic impacts of nitrogen and B_12_ depletion on SAM availability (Table 4). This observation is intriguing and warrants further exploration via SAM metabolite analysis under conditions of varying B_12_ and nitrogen availability.

**Table 4:** **B_12_ and B_1_ related genes from published *P. tricornutum* EST libraries**.

	**Original**	**Si−**	**Si+**	**Low Fe**	**n replete**	**N-starved**	**Urea, low N**	**Ammonia,**	**Description**
	**standard**				**chemostat**			**low N**	
	**os**	**sm**	**sp**	**fl**	**nr**	**ns**	**ua**	**aa**	
**B_12_-RELATED**									
G18319	0	0	1	9	4	15	20	33	s-adenosyl homocysteine hydrolase
G48322	3	2	0	0	1	0	2	3	CBA1
G18665	1	0	1	1	1	3	10	11	Glycine hydroxymethyltransferase
G28056	0	7	11	0	0	2	0	1	MetE
G913.1	1	0	0	5	2	3	0	1	S-adenosylmethionine synthetase
G54015	0	0	1	4	1	6	0	0	Glycine hydroxymethyltransferase
G23399	1	0	0	9	0	1	5	5	MetH
G51830	4	0	3	0	0	3	7	4	Methylmalonyl co a mutase
G30471	0	0	1	0	0	0	4	2	Methylenetetrahydrofolate reductase
**B_1_ USE**									
G20183	2	0	1	0	0	2	0	0	Transketolase
G20360	0	0	0	0	0	3	0	0	Pyruvate dehydrogenase e1 component beta subunit
G12375	0	0	0	0	0	2	1	0	Pyruvate dehydrogenase e1 component alpha subunit
G29016	2	0	0	0	0	4	0	2	2-oxoglutarate dehydrogenase e1 oxoglutarate alpha-ketoglutaric
G37341	2	1	3	0	0	0	2	7	Acetolactate synthase
G48444	0	0	1	0	0	1	1	1	2-oxoglutarate dehydrogenase e1 component
G46387	0	0	0	0	1	0	0	0	Dehydrogenase, E1 component
G36257	0	0	0	0	0	0	0	1	Fructose-6-phosphate phosphoketolase
G9476	1	2	4	2	0	1	0	0	2-oxoisovalerate dehydrogenase alpha, mitochondrial expressed
G41856	14	0	2	1	2	3	12	3	Plastid transketolase
G29260	5	0	0	2	1	2	11	6	Probable transketolase
G11021	0	3	2	3	1	0	0	0	Branched-chain alpha-keto acid decarboxylase e1 beta subunit
**B1 SYNTHESIS**									
G34373	0	0	0	1	0	0	0	0	Possible ThiF
G1689.1	3	0	0	2	2	5	1	0	Possible Dsx
G31544	4	2	0	1	0	4	3	2	Possible ThiO
G38085	9	1	0	0	1	1	3	4	ThiC
G47293	0	0	0	0	0	0	0	0	Possible ThiD/E
G5423	1	0	0	0	0	0	0	0	TPK
**LCPA BIOSYNTHESIS**									
G7617	0	0	1	0	0	3	4	0	s-adenosylmethionine decarboxylase proenzyme
G7910	0	0	0	0	0	0	0	1	Spermine synthase
G3362	0	2	0	0	0	4	9	0	S-adenosylmethionine decarboxylase
G7621	0	0	0	0	0	0	1	0	s-adenosylmethionine decarboxylase proenzyme

Treatment descriptions and labels can be found in Maheswari et al. (2010).

In addition, nitrogen limitation has been previously identified as an important factor driving DMSP and DMS produced by phytoplankton populations. Nitrogen deprivation, more than any other nutrient starvation scenario tested, led to enhanced DMSP production per cell in an important oceanic diatom (Bucciarelli and Sunda, 2003; Sunda et al., 2007). A possible explanation for this trend is that under nitrogen starvation, N-containing osmolytes such as proline, homarine, and GBT are replaced by DMSP, which does not contain nitrogen (Bucciarelli and Sunda, 2003). There is some evidence that under N-replete conditions, GBT and homarine replace DMSP in *T. pseudonana* and that GBT concentrations increase upon addition of N to N-starved cultures of diatoms and coccolithophores (Keller et al., 1999). If DMSP is in fact used to replace N-containing osmolytes, B_12_ starvation coupled with N-limitation has the potential to negatively impact that substitution in at least two ways. The first is by potentially limiting the amount of DMSP produced due to restricted methionine availability. The second is again through SAM deprivation, which has been hypothesized to play an important role in diatom metabolism under low B_12_ conditions (Figure 3; Bertrand et al., 2012). These metabolic connections suggest that there may be synergistic impacts of B_12_ and N starvation on DMSP-producing algal strains. Alternatively, if the primary function for DMSP is as an antioxidant, increases in DMSP as a function of nitrogen starvation could be due to elevated demand for DMSP under the oxidative stress induced by nitrogen deprivation (Sunda et al., 2002, 2007). If DMSP in fact serves an important antioxidant role and if thiamine is shown to be an important algal antioxidant as well, this suggests that there could be potentially important interactions between B_1_, B_12_, and N availability in algal cells in response to oxidative stress.

Synthesis of GBT, in many organisms, also requires SAM as a methyl group donor. Also like DMSP, there is evidence that GBT production is tied to nitrogen metabolic status of individual cultures, (Keller et al., 1999, 2004). Since GBT synthesis requires nitrogen and is likely SAM dependent, B_12_ starvation may prompt substitution of other osmolytes, such as proline, for GBT as well as DMSP. This may have important implications for cellular nitrogen cycling. Notably, proline is generated from ornithine via activity of ornithine cyclodeaminase. Ornithine is an important metabolite in the urea cycle, which is a major pathway for nitrogen recycling in diatoms and potentially other algae (Fernie et al., 2012). If the proline balance were significantly impacted as a result of a metabolic cascade resulting from changes in the osmolyte balance, this could have significant impacts on overall cellular nitrogen metabolism.

### B_12_ and B_1_ starvation impacts on amino acid and polyamine biosynthesis

The major organic constituent of diatom silica frustules are a series of long chain polyamines (LCPAs). Different diatoms synthesize different suites of LCPAs (Kroger et al., 2000). These molecules, along with silica deposition proteins called silafins and silafin-like girdle band and nanopattern-associated proteins call cingulins (Scheffel et al., 2011), induce biomineralization, and are responsible for differences in frustule morphology between diatom groups. LCPAs vary in chain length and degree of methylation, but appear to all be synthesized from putricine, spermidine, or spermine precursors. These precursors are synthesized sequentially from ornithine, with spermidine, and spermine production both requiring SAM as a propylamine donor. Subsequent steps in LCPA formation likely require SAM as well (Kroger and Poulsen, 2008). LCPAs recovered in net tows are mostly putracine-based, with varying degrees of methylation, suggesting that SAM is an important component of LCPA production for field diatom populations as well (Bridoux et al., 2012). Conceivably, reduced SAM production through B_12_ starvation could induce changes in silica frustule formation by decreasing the pool of available LCPAs. Indeed, reduction of LCPA production as a result of the addition of an inhibitor for ornithine decarboxylase, which is known to be involved in polyamine biosynthesis, dramatically reduced biogenic silica formation in *T. pseudonana* (Frigeri et al., 2006). In diatoms, possible LCPA biosynthesis genes have been identified. These are potentially gene fusions of bacterially derived polyamine biosynthetic enzymes S-adenosylmethionine decarboxylase (SAM DC) and an aminopropyltransferase (Michael, 2011), which require input of SAM. The Met salvage pathway would need to be efficient, and if SAM starvation does result from B_12_ deprivation, there could be substantial implications of low B_12_ for LCPA biosynthesis. Ornithine represents a significant component of the carbon and nitrogen pool within phytoplankton cells and is a centrally important metabolite in the ornithine urea cycle (OUC), which is the major distribution hub for nitrogen in diatom cells (Allen et al., 2011; Bender et al., 2012). If SAM starvation results in major changes in ornithine balance through alterations in polyamine biosynthesis, this would hold substantial ramifications for the impact of B_12_ deprivation on nitrogen cycling.

Overall, it seems that N starvation could induce up-regulation in pathways that demand B_12_, such as methionine and SAM synthesis. This would potentially be reflected in elevated expression of B_12_ acquisition proteins under nitrogen limitation, and elevation of proteins required to generate methionine.

### Links between N, B_1_ and B_12_ through sulfur metabolism in eukaryotic phytoplankton

Connections between nitrate reduction and sulfur assimilation are well known. Sulfate reduction is thought to be regulated by nitrogen nutrition in plants (Koprivova et al., 2000; Takahashi et al., 2011); this may also be true for phytoplankton, as sulfur uptake and assimilation genes in diatoms appear to be responsive to nitrogen availability. Both B_1_ and B_12_ have important ties to sulfur metabolism, since B_12_ is important for sulfur amino acid cycling and DMSP synthesis and B_1_ is produced from thiazole, a sulfur containing moiety. Indeed, in plants, methionine synthesis and other aspects of sulfur metabolism are very tightly regulated by SAM availability. If the B_12_ dependence of SAM availability hypothesized for phytoplankton is verified (Bertrand et al., 2012), this suggests that B_12_ availability may influence additional aspects of sulfur and nitrogen metabolism.

### Influence of B_12_ and B_1_ starvation on the diatom urea cycle through impacts on the citric acid cycle and amino acid cycling

Important impacts of vitamins on amino acid and amine cycling include the previously discussed impact of B_12_ on cysteine and methionine cycling and the impact of B_1_ on branched amino acid synthesis. B_1_ contributes to the first step in valine synthesis as well as important steps in amino acid degradation and recycling via keto acid dehydrogenase activity (Binder et al., 2007). In addition, B_1_ appears to impact nitrogen assimilation and amino acid recycling though the dependence of 2-oxoglutarate dehydrogenase (OGDHC) on the cofactor (Bunik and Fernie, 2009). For instance, potato OGDHC inhibition causes reductions in nitrate assimilation as well as increases in glutamate and GABA accumulation (Araujo et al., 2008). This suggests that disturbances in B_1_ metabolism may have profound affects for nitrogen assimilation and amino acid recycling, though this has yet to be confirmed for phytoplankton.

The OUC is of central importance to diatoms and potentially other phytoplankton as a nitrogen assimilation and repackaging hub. The OUC and the citric acid (TCA) cycles are linked (Allen et al., 2011). Mitochondrial amino acid catabolism yield carbon skeletons for the TCA cycle as well as ammonia and bicarbonate that is shunted into the OCU. This connection is supported by metabolic data suggesting that fumarate and malate, important TCA cycle intermediates, display similar patterns as OUC metabolites in diatom cell lines with altered urea cycle pathways (CPS knockdowns; Allen et al., 2011). Both B_12_ and B_1_ play important roles in the citric acid (TCA) cycle. For example, B_12_ is a cofactor for mmcM which generates succinyl coA from methylmalonyl coA, an amino acid degradation product. Expression of the gene encoding mmcM is upregulated under—N conditions in *P. tricornutum* EST libraries (Table 4), suggesting that there could be consequences of reduced mmcM activity for cells experiencing nitrogen deprivation. B_12_ availability does not appear to influence mmcM expression (Bertrand et al., 2012). It is notable that mmcM expression levels are not insignificant in diatom transcriptome studies, suggesting that this gene product may be of utility to phytoplankton despite the fact that the presence of this gene in phytoplankton genomes does not confer an absolute B_12_ demand (Table 1). There are numerous connections between B_1_ and the citric acid cycle. B1 is required for the generation of acetyl CoA from pyruvate via the pyruvate dehydrogenase complex. The enzyme ODG is also thiamine-dependent and plays a important role in the citric acid cycle. This enzyme is also thought to be a important player in plant nitrogen assimilation though its impact on glutamine stores (Bunik and Fernie, 2009). Interestingly, the reactant consumed by this protein, 2-oxoglutarate, accumulates strongly in diatom cell lines with impacted urea cycle (Allen et al., 2011). These data suggest that B_1_- and B_12_-dependent metabolisms play key roles in steps that maintain cellular carbon and nitrogen recycling; synergistic impacts of B vitamin deprivation and N starvation are therefore likely.

## Synthesis and implications for eukaryotic phytoplankton ecology

**Figure 4:**
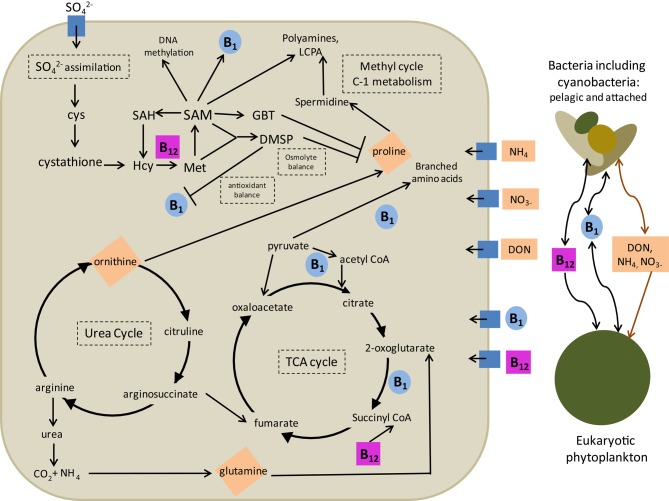
**An overview of B_12_ and B_1_ interactions with nitrogen metabolism in eukaryotic phytoplankton.** Four major intracellular mechanisms are outlined: (1) impacts on osmolyte and antioxidant production and utilization and (2) impacts on polyamine biosynthesis via the methyl cycle, (3) impacts on the urea cycle and amino acid recycling through impacts on the citric acid cycle, and (4) impacts of nitrogen balance on sulfur assimilation. Major cellular nitrogen stores impacted by B_1_ and B_12_ availability are shown in orange diamonds. Arrows denote direction of reaction, production, or consumption. Bars denote potential negative feedbacks, where increases in originating compound may decrease abundance or importance of the connected compound. Also described are major interactions with other microbial groups outside the cell in terms of production and consumption of B_1_, B_12_, and nitrogen sources. Groups considered include pelagic and attached bacteria, including cyanobacteria (brown) and other eukaryotic algae (green). Relevant acquisition pathways are denoted by blue boxes. Cys, cysteine; Hcy, homocysteine; SAH, S-adenosyl homocysteine; SAM, S-adenosyl methionine; GBT, glycine betaine; DMSP, dimethylsulfonium propionate.

Many of the interactions between B vitamins and N metabolism described above have the potential to profoundly influence eukaryotic phytoplankton ecology and are summarized in Figure 4. From these interactions, we can hypothesize that nitrogen limitation, experienced by phytoplankton in much of the ocean, may induce enhanced demand for B_12_ and B_1_ via a variety of mechanisms. These include substitution of N-containing osmolytes with DMSP, substituting N-containing antioxidants and DMSP with thiamine, and effectively recycling amino acids and glutamine stores utilizing high amounts of B_1_. There are also interactions discussed above that would result in B vitamin deprivation leading to impaired nitrogen recycling which could conceivably increase nitrogen demand in phytoplankton cells. These include impaired glutamine recycling due to reduced 2-oxogultarate dehydrogenase and pyruvate dehydrogenase activity and impaired ornithine and proline cycling due to B_1_ and B_12_ impacts on the TCA cycle as well as through potential imbalances in the methyl cycle due to B_12_ deprivation. These mechanisms all suggest that biochemical interactions between B vitamin and N limitation have the potential to lead to interactive colimitation and thus that the B vitamin and N colimitations observed in field studies (Koch et al., 2011; Gobler et al., 2007) may be due to both independent secondary limitation or dependent colimitation scenarios (Saito et al., 2008).

These biochemical dependencies have the potential to impart changes in phytoplankton species composition based on differences in B_1_ and B_12_ demand between phytoplankton groups. Diatoms are thought to rely on an efficient urea cycle for distributing and recycling nitrogen (Allen et al., 2011; Bender et al., 2012). The impacts of B_12_ and B_1_ deprivation on the efficiency of the urea cycle, therefore, may disproportionately impact diatoms. In addition, if dinoflagellate SAM demand is indeed elevated over other phytoplankton as hypothesized, it is possible that B_12_ deprivation could disproportionately impact dinoflagllate strategies for coping with nitrogen deprivation, such as the use of DMSP to replace N-containing osmolytes. These impacts may be of particular importance to harmful algal bloom species, which are known to have disproportionately high instances of B_1_ and B_12_ auxotrophy (Tang et al., 2010). Additionally, toxin production by some dinoflagellate species has been shown to increase under N-limitation (Ransom Hardison et al., 2012); synthesis pathways of many dinoflagellate toxins such as saxitoxin and brevetoxin are thought to be SAM-dependent (Lin, 2011). Together, these data suggests that HAB species may be more susceptible than others to impacts of these dependent colimitations between N and B vitamins and that these colimitations may additionaly impact toxin production rates. This is further evidence that B vitamin dynamics should be considered when predicting and evaluating potential for harmful algal bloom scenarios.

Given that B_12_, B_1_, and nitrogen availability to eukaryotic phytoplankton all have potential to be impacted by bacterial community composition and activity, the bacterial community is likely an important driver of when and where instances of these dependent colimitations may be important. This may be especially true when considering timing and species composition in spring bloom scenarios, which is an active area of continued research today (Mahadevan et al., 2012). Swift and Guillard (1978) determined that spring bloom diatom species, though not B_12_ auxotrophs, grew faster and experienced shorter lag phases in the presence of the vitamin, suggesting that possible interactions between N and S metabolism, and B_12_ utilization could be important for bloom timing and species composition. Recent work also suggests that bacterioplankton respond to various phases in spring blooms by changing both metabolic potential and species composition over time (Teeling et al., 2012). This could have important impacts for B_12_ and B_1_ production and consumption as well as for nitrogen availability and recycling. Mounting evidence suggests that there could be synergistic interactions of these impacts on eukaryotic phytoplankton that could influence not only species composition but also bloom timing and overall productivity. This suggests that time series measurements, over both day to week and seasonal timescales, which include B_12_, B_1_, and nitrogen species concentration measurements and uptake rates as well as protein or transcript-based indicators of nitrogen and vitamin deprivation, would be useful, particularly in conjunction with bacterioplankton community composition assessments and implementation of B vitamin biosynthesis indicators. Locations where this would be of considerable interest include high latitude ecosystems, which largely lack B_12_ producing cyanobacteria, coastal locations with HAB blooming dinoflagellates, and diatoms as well as the North Atlantic, before during and after bloom scenarios.

### Conflict of interest statement

The authors declare that the research was conducted in the absence of any commercial or financial relationships that could be construed as a potential conflict of interest.
